# Study protocol: prediction of stroke associated infections by markers of autonomic control

**DOI:** 10.1186/1471-2377-14-9

**Published:** 2014-01-13

**Authors:** Dirk Brämer, Heike Hoyer, Albrecht Günther, Samuel Nowack, Frank M Brunkhorst, Otto W Witte, Dirk Hoyer

**Affiliations:** 1 Hans Berger Department of Neurology, Jena University Hospital, Jena, Germany; 2 Institute of Medical Statistics, Computer Sciences and Documentation, Jena University Hospital, Jena, Germany; 3 Integrated Research and Treatment Center, Center for Sepsis Control and Care (CSCC), Jena University Hospital, Jena, Germany; 4 Center for Clinical Studies (CCS), Jena University Hospital, Jena, Germany; 5 Paul-Martini-Clinical Sepsis Research Unit, Department of Anaesthesiology and Intensive Care Medicine, Jena University Hospital, Jena, Germany

**Keywords:** Stroke, Infection, Pneumonia, Autonomic nervous system, Heart rate variability

## Abstract

**Background:**

Infection is the most important complication after acute stroke. This is substantially based on a stroke-induced immunosuppression. Heart rate variability (HRV) represents the autonomic nervous system activity in connection with stroke-induced immunomodulation and infections. We demonstrated in a feasibility study that HRV indices obtained in patients without acute post-stroke infections can predict infections in the subsequent sub-acute phase.

**Methods/Design:**

The study PRED-SEP is a prospective observational study. Adult patients with acute ischemic infarction in the territory of the middle cerebral artery and severe neurological deficit (National Institutes of Health Stroke Scale: NIHSS ≥ 8) are recruited. Primary endpoint is the development of infections, secondary endpoints are SIRS and severe sepsis in the sub-acute phase (day 3–5) after stroke and the functional outcome after 3 months. Infection is defined according to the PANTHERIS study and comprises pneumonia, urinary tract infection and infections without determined focus. SIRS and severe sepsis are defined according to German Sepsis Society guidelines. Functional outcome is measured by lethality and neurological scores (modified Rankin Scale, Barthel Index). Prognostic factors are HRV risk indices calculated from selected intervals of 24 h ECG measurements within 48 hours after symptom onset. It is planned to recruit 240 patients.

HRV risk indices (predictors) will be calculated according to standards and procedures previously developed and published by the authors. The predictive effects of HRV indices on infections will be estimated by fitting logistic regression models and estimating odds ratios with 95% confidence intervals. A prespecified modelling procedure will be applied to estimate unadjusted and confounder adjusted odds ratios. Secondary endpoints will be analysed in the same way. The functional outcome scales will be dichotomized. The association between HRV indices and pro- and anti-inflammatory markers will be quantified by calculating the appropriate correlation coefficients according to scale (Person or Spearman).

**Discussion:**

Since a general prophylactic antibiotic treatment after stroke is not recommended, results of this study could have essential implications for an early identification and hence, timely appropriate treating of stroke-induced infections.

**Trial registration:**

Prädiktoren für die Sepsis - Pred Sep, German Clinical Trials Register: DRKS00003392.

## Background

Stroke-induced infection is the most relevant complication after acute ischemic stroke with further implications of developing severe sepsis and adverse outcome. Most relevant stroke-induced infections are pneumonia (4-22%) and urinary tract infections (UTI, 6.3-30.5%) [1]). Both of which are associated with poor 3 months functional outcome (modified Rankin Scale score 3–6), namely pneumonia with OR = 4.4 (2.2-9.0) and UTI with OR = 2.7 (1.3-5.6) [2]. In multivariate prediction models infection is an independent factor besides age and National Institutes of Health Stroke Scale (NIHSS) for 3 months functional outcome (modified Rankin Scale) [3]. General antibiotic therapy in patients with acute stroke significantly reduced the incidence of infections, but only non-significantly reduced the number of patients who died [4]. Preventive post-stroke antibiotic therapy is not recommended according to guidelines [5]. Therefore, early antibiotic therapy is recommended as soon as an infection is identified [6] and early markers of post-stroke infections are needed.

The analysis of the activity of the autonomous nervous system (ANS) may provide a novel timely appropriate approach for the early detection of developing infections. Stroke-induced immunosuppression mainly results from activation of sympathetic mediated pro-inflammatory cytokine production. In contrast, vagus nerve releases acetylcholine which inhibits the production of pro-inflammatory cytokines, but maintains production of anti-inflammatory cytokines [7-10]. Those mechanisms are part of the “Central nervous system injury-induced immune deficiency syndrome” mediated by the sympathetic nervous system, the N. vagus (parasympathetic nervous system), and the hypothalamo–pituitary–adrenal (HPA) axis [11]. After acute ischemic stroke overall heart rate variability (HRV) is depressed, while the sympathetic part dominates in connection with the reduced vagal HRV (e.g. [12-14]).

In patients without any kind of brain damage HRV indices were correlated with inflammation markers. Higher levels of Interleukin 6 (IL-6) and C-reactive peptide (CRP) were typically associated with increased heart rate, reduced overall HRV, and reduced sympatho-vagal balance [15-19]. In septic patients severity of illness was associated with reduced sympatho-vagal balance indices [20]. Sepsis was predictable by reduced overall HRV [21]. In patients with sepsis and multiple organ dysfunction syndrome reduced VLF (very low frequency power) was associated with a lower survival rate. In those patients the discriminatory value of altered sympatho-vagal control was strongest reflected by HRV complexity indices based on autonomic information flow (AIF) [22-24].

In a feasibility study we enrolled prospectively forty-three patients with acute ischemic stroke. The predictability of sub-acute infections (day 4 ± 1 after admission) was investigated in 34 patients without acute infection by means of HRV indices obtained in the acute period (48 h after admission). Associations between HRV indices in the acute period and post-stroke infection in the sub-acute period were examined. Sub-acute infection could be predicted in patients without clinical or paraclinical (white blood cell count (WBC) and CRP) signs of infection in the acute period by: 1) an increased HFnorm (normalized high frequency power), but a reduced LFnorm (normalized low frequency power) as well as a reduced LF/HF ratio during the day for all parameters, and 2) a reduced LF (low frequency power) and VLF (very low frequency power) at night (all changes p < 0.05) [25].

HRV indices are readily available from routine stroke monitoring systems. They were found to be associated with sub-acute post-stroke infections. Preceding the analysis of routine blood samples HRV analysis probably allows an early diagnosis of a developing infection. Thus, an HRV-based early diagnosis of post-stroke infection should be investigated in more detail. The method may have the potential of a novel tool to support decisions about a timely and appropriate treatment.

### Objectives

The primary objective of this study is the prediction of stroke-induced infections (primary endpoint) by means of HRV indices. Secondary endpoints are SIRS, severe sepsis, and functional outcome. In further analyses, correlations between HRV indices and blood markers will be explored and novel innovative HRV indices will be explored.

## Methods/Design

### Study design

The study is a prospective observational cohort study at the Hans Berger Department of Neurology, University Hospital Jena.

### Study population

*Inclusion criteria:*

– Patients with ischemic infarction in the territory of the middle cerebral artery with or without insular cortex involvement

– Age ≥ 18

– NIHSS ≥ 8

*Exclusion criteria:*

– Ongoing infections at admission

– History of previous strokes

– Cardiogenic arrhythmias

### HRV indices for prediction of outcomes

The key HRV risk indices are chosen according to the results of our feasibility study. HFnorm and LFnorm (markers of sympatho-vagal adjustment) are calculated from one defined 3 h interval during day time and VLF (integrative marker of autonomic and humoral control) is calculated from one defined 3 h interval during night time, both selected from the 24 h Holter ECG recordings that started within 24 h hours after onset of stroke symptoms.

### Outcome measures

*Primary endpoint:*

**Infection** defined according to the PANTHERIS study [26]: I) pneumonia was defined as presence of at least one of the A and one of the B criteria: A) abnormal respiratory examination, pulmonary infiltrates in chest X-Ray; B) productive cough with purulent sputum, microbiological cultures from lower respiratory tract or blood cultures, leukocytosis, and elevation of CRP. II) Urinary tract infection was defined as the presence of two of the following criteria: fever (>38.0°C), urine sample positive for nitrite, leucocyturia, and significant bacteriuria. III) Infection was also diagnosed if temperature was above 38.0°C on at least 2 determinations with additional leucocytosis and positive blood cultures, but no determined focus.

*Secondary endpoints:*

– **SIRS:** criterium II according to Guidelines of the German Sepsis Society

– **Severe sepsis***:* criteria I, II and III according to Guidelines of the German Sepsis Society

– **Functional outcome**: day 5 and 3 months after insult (modified Rankin Scale, Barthel Index)

The cumulative incidence of infections, SIRS or severe sepsis will be estimated up to day 5 after the onset of stroke.

Guidelines of German Sepsis Society

I. **Confirmation of infection**

• Diagnosis of an infection on the basis of microbiological evidence or clinical criteria

II. **Systemic inflammatory host response (SIRS) (at least 2 criteria)**

• Fever (**≥**38°C) or hypothermia (**≤**36°C) confirmed by rectal, intravascular or intravesical measurement

• Tachycardia: heart rate **≥**90 bpm

• Tachypnea (frequency **≥**20/min) or hyperventilation (PCO2 **≤**4.3 kPa/ **≤**33 mmHg)

• Leukocytosis (**≥**12000/mm3) or leukopenia (**≤**4000/mm3) or **≥**10% immature neutrophils in differential blood count

III. **Acute organ dysfunction (at least 1 criterion)**

• Acute encephalopathy: reduced alertness, disorientation, agitation, delirium

• Relative or absolute thrombocytopenia: decrease in platelet counts by more than 30% within 24 hours or a platelet count of less than 100.000/mm3. Thrombocytopenia due to acute hemorrhage or immunological causes must be ruled out.

• Arterial hypoxemia: PaO2 **≤**10 kPa (**≤**75 mmHg) while breathing ambient air or a PaO2/FiO2 ratio of **≤**33 kPa (**≤**250 mmHg) on oxygen administration. A clinically manifest heart or lung disease must be ruled out as a cause of hypoxemia.

• Renal impairment: diuresis of **≤**0.5 ml/kg/h for at least 2 hours despite adequate volume resuscitation and/or an increase in serum creatinine level to > twice the upper limit of normal (ULN).

• Metabolic acidosis: Base excess of ≤ -5 mmol/L or lactate concentration of > 1.5×ULN.

### Further analysis

Correlation analysis between HRV indices (LFnorm, HFnorm, VLF) and pro- and anti-inflammatory molecular markers (copeptine, PCT, CRP, IL-6, IL-10, mHLA-DR, TNF alpha) daily taken at day 1 to day 5.

The predictive value of Autonomic Information Flow (AIF) indices, which are innovative markers of complex autonomic control extending the established HRV indices, will be explored based on previous developments and studies [23,24,27,28].

### Key data acquisition and individual time line

Patients are enrolled on day 1 after admission (at most 24 hours after symptom onset). The following baseline data are documented: demographic data, medical history, medications prior to admission, vital signs (including blood pressure, NIHSS), stroke characteristics such as symptom onset as well as ischemia location and size obtained by cranial MRI or CT, blood parameters (CRP, WBC) at admission to the emergency unit.

For the HRV analysis a 24 h-Holter-ECG recording is started on the morning after the stationary admission around 9 h00 (at most 24 h hours after symptom onset). A second 24 h-Holter-ECG is applied on day 5.

Up to day 5 the neurological status (NIHSS, Rankin Scale, Barthel-Index), clinical signs of infection (fever, pathological findings of examination) and blood parameters (CRP, WBC, PCT, cortisol, ACTH, copeptine, IL-6, IL-10, mHLA-DR, TNFalpha) are documented in a daily visit.

After 3 months patients are interviewed via by telephone contact to record the clinical outcome (Modified Rankin Scale, Barthel-Index).

### Methods against bias

Patients eligible for the study will be recruited consecutively to prevent selection bias. HRV risk indices will be calculated according to standards and procedures previously developed and published by the applicant and blinded to the individual outcome diagnosis of the patients. Potential confounders like age, size of insult, localisation (laterality/insular cortex), use of beta blocker at admission, baseline NIHSS will be collected and included in the prognostic model according to a modelling procedure prespecified in a statistical analysis plan.

### Sample size

In a feasibility study 12 out of 34 patients (about 35%) with appropriate ischemic territorial stroke developed a new infection during day 3 to 5 after onset of stroke symptoms. Daytime LFnorm at baseline as a key HRV parameter was significantly lower in patients with infections compared to those without infections (mean = 40.1, SD = 19.4 and mean = 58.2, SD = 13.6, respectively). From the perspective of prognosis, an increase of LFnorm by one standard deviation (SD = 17.9) was associated with a 0.4 fold chance of getting an infection (OR = 0.4 or ln OR = β = -0.916). The logistic regression test of β =0 (α = 0.05 two-sided) will have 80% power to detect a β of -0.916 (OR = 0.4) with a sample size of 82 patients. To detect this effect while controlling for potential confounders sample size must be multiplied by the variance inflation factor 1/(1-σ^2^) where σ^2^ is the squared multiple correlation of LFnorm with further covariates in the model. A sample size of 136 will be necessary assuming σ^2^ = 0.4 and an infection proportion of 35% (nQuery Advisor 6.01).

According to the feasibility study 43% of patients had to be excluded after enrolment due to cardiogenic arrhythmias or ongoing infections at admission leaving about 57% of patients for the prognostic modelling. Therefore, a total of 240 patients should be allocated to the study.

### Feasibility of recruitment

Out of approximately 740 stroke patients admitted to the Department of Neurology per year about 170 meet the inclusion criteria, hence during 2 years of recruitment about 340 patients are available. Supposing that 70% to 80% of patients will consent to participate about 240 patients could be allocated to the study. These patients do not participate in competing studies.

### Statistical analysis

HRV risk indices (predictors) will be calculated from selected intervals of 24 h ECG measurements according to standards and procedures previously developed and published by the applicant. The predictive effects of HRV indices on infections will be estimated by fitting logistic regression models and estimating odds ratios with 95% confidence intervals. A prespecified modelling procedure will be applied to estimate unadjusted and confounder adjusted odds ratios. Secondary endpoints will be analysed in the same way. The functional outcome scales will be dichotomized. The association between HPV indices and pro- and anti-inflammatory molecular markers will be quantified by calculating the appropriate correlation coefficients according to scale (Person or Spearman).

### Ethical considerations

All stroke patients involved in this study are treated at the stroke unit according to the guidelines of the German Stroke Society (DSG) and German Neurological Society (DGN). Standard ECG measurements are non-invasive and without any risk for the patients. It is part of the standard medical assessment and is easily done at bedside. Laboratory investigations are without risk for the patient due to the minimal quantities required. The protocol was approved by the ethics commission of the University Hospital Jena. A written informed consent is obtained from all patients or their legal representatives. According to the ethics commission vote the written informed consent can be obtained retrospectively after the acute stroke period. In cases of decline the study data are deleted.

The study was registered at the German Clinical Trials Register (DRKS00003392).

### Recruitment

The recruitment, observation and follow-up of patients started in February 2012. All study related activities follow the study protocol and an elaborated study manual. It is planned to finish recruitment in May 2014.

## Discussion

The goal of this study is to show that HRV indices obtained in the acute post-stroke period are able to predict infections in the subsequent sub-acute period. HRV indices represent the role of ANS activity in connection with immunomodulation and developing infections after stroke. They would be readily available from routine ECG stroke monitoring systems. Their continuous consideration would allow an earlier identification and, hence, timely appropriate treatment of developing infections.

Recently, the clinical A^2^DS^2^ score to predict pneumonia in acute ischemic stroke was developed and validated using data of two independent stroke registers. This 10-point risk score (points) includes age ≥75 years (1), atrial fibrillation (1), dysphagia (2), male sex (1), stroke severity according to the National Institute of Health Stroke Scale 0–4 (0), 5–15 (3), ≥16 (5) [29].

Biomarkers obtained from post-stroke blood samples, like WBC, CRP, PCT, and copeptin were all independent predictors of pneumonia, UTI, and other infections [30]. Those authors concluded that “the combination of established inflammatory makers (WBC, CRP) combined with a biomarker of stress (copeptin) or a biomarker of bacterial infection (PCT) probably reflects better the complexity of an infection than one biomarker alone and may lead to a more accurate prediction of a beginning but not yet clinically apparent infection”.

In addition to the HRV indices all components of the A^2^DS^2^-score and the above mentioned blood markers are recorded in our study. We will be able to put our HRV study results into perspective by analysing several potential predictors and, depending on the number of observed outcome events, their combinations in multiple regression models.

## Abbreviations

ANS: Autonomous nervous system; CT: Computer tomography; HPA: Hypothalamo–pituitary–adrenal; MRI: Magnetic resonance imaging; NIHSS: National institutes of health stroke scale; OR: Odds ratio; SD: Standard deviation; SIRS: Systemic inflammatory response syndrome; UTI: Urinary tract infections.; AIF: Autonomic information flow; ECG: Electrocardiogram; HRV: Heart rate variability; HF: High frequency power; HFnorm: Normalized high frequency power; LF: Low frequency power; LFnorm: Normalized low frequency power; VLF: Very low frequency power.; ACTH: Adrenocorticotropic hormone; CRP: C-reactive peptide; IL-6: Interleukin 6; IL-10: Interleukin 10; HLA-DR: Monocytic Human Leukocyte Antigen-DR; PCT: Procalcitonin; TNFalpha: Tumor necrosis factor alpha; WBC: White blood cell count.

## Competing interests

The authors declare that they have no competing interests.

## Authors’ contributions

DH, HH, AG, and DB FMB and OW developed the concept and design, HH as study statistician developed the statistical design, DB and AG are the trial physicians, DB and SN acquire the data, DH, HH, DB, and SN will analyse the data. All authors read and approved the final manuscript.

## Pre-publication history

The pre-publication history for this paper can be accessed here:

http://www.biomedcentral.com/1471-2377/14/9/prepub
